# An Etiological Investigation of Paraneoplastic Cerebellar Degeneration in Ovarian Cancer Patients: A Systematic Review

**DOI:** 10.7759/cureus.31154

**Published:** 2022-11-06

**Authors:** Akbar A Fidahussain, Ali Abid, Awais A Paracha, Varun E Jeevan, Joseph Rueve, Mckimmon Engelhardt, Cody Schrock, Sofia Ghani, Hari K Nair

**Affiliations:** 1 Biomedical Engineering, Saint Louis University, St. Louis, USA; 2 Biology, Saint Louis University, St. Louis, USA; 3 Hematology and Oncology, Saint Louis University School of Medicine, St. Louis, USA; 4 Neurology, University of Missouri School of Medicine, Columbia, USA; 5 Neuroscience, Saint Louis University, St. Louis, USA

**Keywords:** autoimmune response, neurological manifestations, gait abnormalities, dysarthria, serous carcinoma, onconeural antibody, malignancy, anti-yo, ovarian cancer, paraneoplastic cerebellar degeneration

## Abstract

Paraneoplastic syndromes (PNS) are uncommon, distinct clinical complications of a primary tumor. Paraneoplastic cerebellar degeneration (PCD) is a PNS that is described as an autoimmune response targeting Purkinje cells in the cerebellum. Ovarian cancer (OC) is one of the most prevalent causes of cancer-related deaths in women. Anti-Yo is the most common onconeural antibody produced in the PCD immune response and is most typically found in ovarian and breast cancer patients. While the current literature highlights the predisposing genetic factors, diagnostic workflows, and treatment options, the pathophysiology of PCD, among other considerations, remains largely unestablished. This review aimed to systematically observe procedural solutions to facilitate an early diagnosis and improve the prognosis of patients with OC-associated PCD. To that end, we examined literature published from 01/01/2015-11/10/2022 indexed in PubMed by using the keywords “paraneoplastic, cerebellar degeneration” combined with “ovarian cancer.” Inclusion criteria were met if PCD and OC diagnoses were made and if studies provided adequate patient information. After screening and assessing records for eligibility using the inclusion and exclusion criteria, 18 articles involving 102 patients were included. The typical patient observed in this sample was diagnosed with International Federation of Gynecology and Obstetrics (FIGO) Stage III, high-grade serous carcinoma. The diagnostic workup typically included a clinical evaluation for dysarthria (50%), ataxia (60%), and gait abnormalities (50%), along with multiple imaging modalities and serological findings (90%). Genetic screening for human leukocyte antigen (HLA) haplotype susceptibility for PCD and immune tolerance modulators regulation may also be recommended prior to starting treatment. Findings support the use of corticosteroids (35%) and intravenous immunoglobulin (IVIg) (40%) as viable treatment options for managing PCD in conjunction with systemic therapy for the primary malignancy. A diagnosis of PCD should be considered if a patient has had a malignancy in the past five years with the presence of explicit cerebellar symptoms. This clinical diagnosis can be further supplemented by serologic and radiologic findings. Recognizing PCD symptoms and scheduling genetic and proteomic testing may help with early diagnosis and better prognosis.

## Introduction and background

Paraneoplastic syndromes (PNS) are a diverse set of clinical complications that occur as a consequence of several primary malignancies. These complications are largely caused by the production of cytokines, hormones, or peptides by the tumor cells or due to an immune response elicited by the primary tumor [1]. Various distinct paraneoplastic syndromes have been reported, including­ dermatological (vasculitis, myositis), rheumatological (polymyalgia rheumatica, hypertrophic osteoarthropathy), endocrinological (syndrome of inappropriate antidiuretic hormone secretion, Cushing’s syndrome, hypercalcemia), neuromuscular (myasthenia gravis, Lambert-Eaton syndrome), and neurological (encephalitis, opsoclonus-myoclonus, and subacute cerebellar degeneration) [1]. Paraneoplastic cerebellar degeneration (PCD) is a rare complication of certain malignancies affecting less than 1% of all cancer patients. It is commonly seen in breast cancer and pelvic malignancies but has also been reported in Hodgkin’s lymphoma, gastric cancer, prostate cancer, and small-cell lung cancer [2,3].

The clinical presentation of PCD includes altered gait, diplopia, and difficulty with fine motor skills, with eventual progression to limb and truncal ataxia [4,5]. These symptoms usually occur over several weeks but can progress rapidly in certain cases [6]. The pathophysiology of PCD is hypothesized to be from antibodies produced in response to an onconeural antigen; this antigen is the cerebellar degeneration-related protein 2 (CDR2) and is expressed by tumor cells [3]. This antigen is also found in the Purkinje cells of the cerebellum. The etiology of PCD is thought to be a cross-reactive immune reaction where antibodies targeted at antigens present in the tumor cells attack the same antigens present in the cerebellum [3]. However, the presence of these antibodies on serologic testing is not essential for diagnosing PCD [7]. Graus et al. (2004) reported that a PCD diagnosis requires less than three months of cerebellar symptoms (ruling out chronic processes) along with a normal brain MRI (ruling out other chronic causes of cerebellar atrophy) in conjunction with a score of 3 on the modified Rankin Scale [8]. Additionally, this must concur with symptoms of ataxia and the diagnosis of cancer within five years of symptom onset [3,8]. As stated earlier, although the presence of antibodies is not required, it can support a diagnosis of PCD. Anti-Yo is the most common antibody that occurs primarily in breast and ovarian cancer (OC) patients [3]. This systematic review focuses specifically on PCD in patients with OC. OC is the fifth most common cause of death in women, with 14,000 deaths annually and a five-year survival rate of 48.6% among patients worldwide [9,10]. In addition, symptoms of OC are non-specific and medical attention is frequently not sought until the disease is in an advanced stage. Typical symptoms include gastrointestinal disturbances such as nausea, bloating, abdominal distention, and early satiety, as well as other symptoms such as dysuria, back pain, dyspareunia, and cachexia [11]. With cerebellar degeneration being a visible PNS that develops months to years before typical OC symptom onset, PCD can serve as a valuable tool in the early diagnosis of ovarian malignancies [3].

PCD is a well-established indicator of occult malignancy. Peterson et al. (1992) reported that in 34 out of 55 patients with PCD studied, the diagnosis of a neoplasm was preceded by symptoms of neurological origin [6]. Furthermore, in all but one of the 19 patients with gynecological cancer, malignancy preceded the evidence of the onset of neuropathy [12]. Identifying PCD requires a multitude of criteria, including the presence of neurological symptoms. The discovery of PCD should be accompanied by an immediate investigation of the primary malignancy, and the discovery of associated onconeural antibodies could indicate an underlying malignancy. Imaging studies alone cannot be used for a conclusive diagnosis of PNS; however, they are essential in ruling out other diagnoses. Surgical removal of the primary tumor is likely the most effective treatment, although it is not effective in all cases [5]. The previous literature highlights key diagnostic features, clinical presentation, and pathology of PNSs through onconeural antibody identification and malignancy screening [5]. However, with established diagnostic criteria for neuronal surface antibodies syndrome (NSAS) and improved diagnostic tools, the incidence of PCD has risen in the last few years [5]. This review aims to develop a complete clinical profile of PCD and propose a diagnostic framework while evaluating potential treatment modalities. By including genomics, radiologic findings, and the complete clinical picture, a provider can effectively narrow down a PCD diagnosis.

## Review

Methods

Literature Search

This systematic review followed the Preferred Reporting Items for Systematic Review and Meta-Analysis Protocols (PRISMA-P) [13] and is registered with the National Institute for Health Research (PROSPERO). Reviewers examined published studies from 01/01/2015 to 11/10/2022, using PubMed as the sole search database. The following search queries were used to elicit articles of relevancy: ((paraneoplastic) OR (cerebellar degeneration)) AND (ovarian cancer).

Study Selection

Three reviewers (A.A., A.F., J.R.) assessed a list of relevant articles to guarantee that the inclusion criteria were met. The following study designs were included: case reports, case series, and retrospective and prospective cohort studies. For inclusion, the study had to describe the findings of patients with a known PCD and OC diagnosis. The study also had to provide evidence of significant cerebellar dysfunction in cases where the patient was diagnosed with a non-specific PNS. In studies with a sample size >1, data were only gathered for the subsets of patients reporting PCD and OC.

Data Extraction

The following data were collected from each study: study design, presence of anti-Yo antibodies, sample size, the mean age of the sample, prognosis, patient history, treatments, imaging modalities, the time between the onset of cerebellar dysfunction and tumor identification, histological subtype, International Federation of Gynecology and Obstetrics (FIGO) staging, neurological assessment, explorative surgery performed, and laboratory results. Statistical analysis was not performed due to the heterogeneity of the included studies. This review was analyzed purely from a thematic perspective with a report on frequencies of items of interest. The following themes were examined: patient characteristics, clinical manifestation, diagnostic workup, treatment regimens, protein expression, and genetic predisposition.

Results

**Figure 1:**
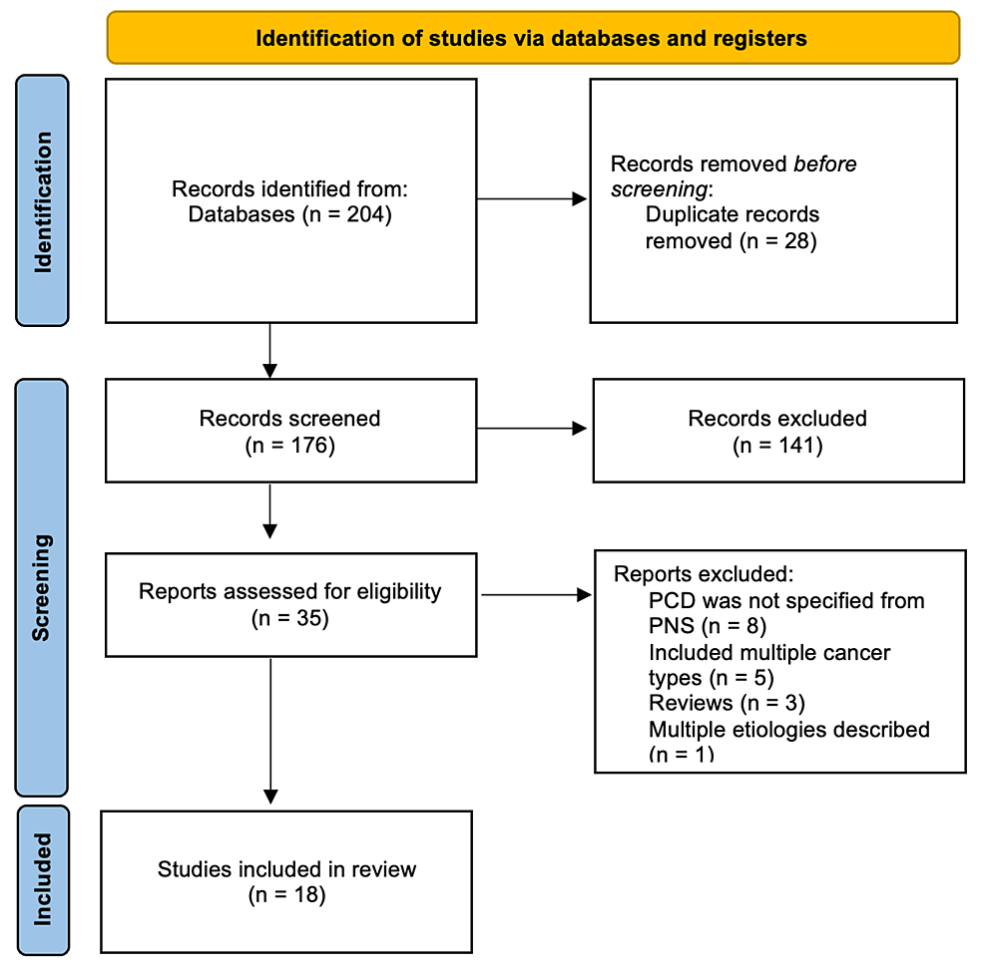
PRISMA 2020 flow diagram depicting the selection of studies PRISMA: Preferred Reporting Items for Systematic Reviews and Meta-analyses; PCD: paraneoplastic cerebellar degeneration; PNS: paraneoplastic syndromes

The study selection procedure is outlined in Figure 1. The search strategy yielded 176 unique records. After the abstract and full-text screening, 18 publications, including 102 cases, were selected. Table 1 presents a systematic summary of the included studies. 

**Table 1:** Summary of studies evaluating patients with paraneoplastic cerebellar degeneration and ovarian cancer PCD: paraneoplastic cerebellar degeneration; CT: computed tomography; MRI: magnetic resonance imaging; IVIg: intravenous immunoglobulin; N/A: not available

Author	Design	Anti-Yo antibody presence	Sample	Mean age (years)	Prognosis	Patient history	Treatments	Imaging modalities used in ovarian cancer or PCD diagnosis
Birch et al. [4]	Case study	Negative	1	73	Worsened impairment	No history of alcohol, smoking, or familial predisposition. The patient had a history of tuberculosis and hypertension	The patient declined treatment beyond tumor resection	CT, ultrasound, echocardiogram, MRI
Shibata et al. [7]	Case study	Negative	1	65	Recovery	No notable medical history	Chemotherapy, IVIg	CT, MRI
Boujoual et al. [14]	Case study	Not tested	2	46	Long-term outcomes not reported	N/A	Treatment not specified beyond tumor resection	MRI, CT
Butt et al. [15]	Case study	Negative	1	69	PCD progression prevented	History of hypertension, asthma, gastroesophageal reflux disease, depression, and familial predisposition to cancer	Chemotherapy	CT, MRI
Chien et al. [16]	Case study	Positive	1	44	PCD progression prevented	No history of alcohol, smoking, illicit drugs, or familial predisposition	Plasmapheresis, chemotherapy	MRI, CT
Cui et al. [17]	Case study	Positive	1	65	Recovery	N/A	Chemotherapy, IVIg, corticosteroids	Ultrasound, CT, MRI
Dandapat et al. [18]	Case study	Positive	1	60	Recovery	No history of psychiatric conditions	The patient declined treatment beyond tumor resection	MRI, CT
Deac et al. [19]	Case study	Positive	1	59	Death	The patient's social, familial, and medical history was insignificant	IVIg, corticosteroids, chemotherapy	MRI, CT, ultrasound, X-ray
Jurkiewicz et al. [20]	Case study	Positive (n=2)	3	16	Recovery (n=3)	N/A	N/A	MRI, CT, ultrasound
Lehner et al. [21]	Case study	Positive (n=5)	6	57	Death (n=2), worsened impairment (n=2), recovery (n=1)	History of breast cancer (n=1)	Chemotherapy (n=4), ERK inhibitor (n=1), corticosteroids (n=5), IVIg (n=3), tacrolimus (n=3), plasmapheresis (n=2)	MRI, CT
Li et al. [22]	Case study	Positive	1	37	Recovery	N/A	Chemotherapy	MRI, CT, ultrasound
Liapi and Sarivalasis [23]	Case study	Positive	1	61	PCD progression prevented	History reported was associated with a favorable oncological prognosis	Chemotherapy	MRI, CT
Renjen et al. [24]	Case study	Positive	1	65	Long-term outcomes not reported	No history of alcohol and smoking	Chemotherapy, IVIg	Radiograph, MRI
Smith and Samkoff [25]	Case study	Negative	1	N/A	Recovery	N/A	Broad-spectrum antibiotics, dexamethasone, acyclovir, corticosteroids, IVIg	MRI, CT, ultrasound
Raspotnig et al. [26]	Retrospective observational study	Positive (n=6)	16	67	N/A	N/A	N/A	N/A
Small et al. [27]	Retrospective observational study	Positive (n=26)	26	64	N/A	N/A	N/A	N/A
Vialatte de Pémille et al. [28]	Retrospective observational study	Positive (n=12)	12	62	N/A	N/A	N/A	N/A
Hillary et al. [29]	Retrospective observational study	Positive (n=43)	43	N/A	N/A	N/A	N/A	N/A

Patient Characteristics

A total of 119 patients were observed across the 18 articles included. After removing patients without a conjunct discovery of PCD and OC (n=17), we were left with a total of 102 patients. The patient population's ages ranged from 16 to 85 years, with the mean age being 51 years. The main patient characteristics are summarized in Table 2. Of note, 71% of patients had the histological subtype of high-grade serous carcinoma (HGSC). Most patients were diagnosed with FIGO stage III.

**Table 2:** Characteristics of patients diagnosed with paraneoplastic cerebellar degeneration and ovarian cancer ^a^Characteristics of the total sample, excluding Hillary et al. ^b^Case study subsample, excluding Raspotnig et al., Small et al., Pemille et al., and Hillary et al. ^c^Included chemotherapy agents: carboplatin, paclitaxel, bevacizumab, doxorubicin, cisplatin, and methotrexate HGSC: high-grade serous carcinoma; MRI: magnetic resonance imaging; CT: computed tomography; CDR2: cerebellar degeneration-related protein 2; CDR2L: cerebellar degeneration-related protein 2L; ERK: extracellular signal-regulated kinase; IVIg: intravenous immunoglobulin

		N (%)
Characteristics of total sample (n=62)^a^		
Histological subtype		
	HGSC	44 (71)
	Other	14 (23)
	Not specified	4 (6)
Staging at tumor diagnosis		
	I	5 (8)
	II	6 (10)
	III	31 (50)
	IV	9 (15)
	Not specified	10 (16)
Case study subsample (n=20)^b^		
Neurological assessment		
	Dysarthria	10 (50)
	Dysmetria	7 (35)
	Ataxia	12 (60)
	Gait abnormality	10 (50)
	Labile mood	2 (11)
	Diplopia	4 (20)
	Vertigo	8 (40)
Imaging		
	MRI	19 (95)
	CT	16 (80)
	Ultrasound	8 (40)
	Radiograph	2 (10)
Laboratory results		
	CSF antibody panel	9 (45)
	Serum antibody panel	13 (65)
	Lumbar puncture	10 (50)
	Serum tumor markers (CA-125)	10 (50)
	CDR2/CDR2L	1 (5)
Explorative surgery		
	Laparotomy/laparoscopy	9 (45)
Outcome		
	Death	3 (15)
	Recovery	9 (45)
	Further impairment halted at tumor resolution	3 (15)
	Worsened impairment	3 (15)
	Not specified	2 (10)
Treatment		
	Chemotherapy^c^	12 (60)
	ERK inhibitor	1 (5)
	Plasmapheresis	3 (15)
	Corticosteroids	7 (35)
	IVIg	8 (40)
	Tacrolimus	3 (15)
	Methylprednisolone	3 (15)
	No further treatment beyond surgical resection	2 (10)
	Not specified	4 (20)

Diagnostic Evaluation

Among the 102 cases analyzed, a diagnostic process was identified in 20 [4,7,14-25]. Diagnostic measures observed in these studies were grouped into neurological assessments, imaging modalities, laboratory findings, and explorative surgeries performed. Neurological assessments were typically conducted initially and included evaluation for dysarthria (50%), dysmetria (35%), ataxia (60%), gait abnormalities (50%), diplopia (20%), and vertigo (40%). These cases were followed up with several imaging methods, such as MRI (95%), CT (75%), or ultrasound (40%) to detect cancerous growth or indications of cerebellar dysfunction. All 20 cases where a diagnostic process was identified reported the use of multiple imaging modalities in the diagnostic workup, the most frequent pairing being an MRI with an abdominal CT. A serum or cerebral spinal fluid (CSF) paraneoplastic panel was obtained in 18 out of 20 patients [4,7,15-23,25]. Serum tumor markers, notably cancer antigen (CA) 125, were tested in half of these patients to confirm the presence of a tumor, track tumor growth, or establish remission status [4,7,15-19,22-24].

Treatment

Fourteen articles included treatment regimens for OC patients with PCD. The most common form of treatment (60%) was combination chemotherapy regimens including carboplatin, paclitaxel, bevacizumab, doxorubicin, cisplatin, and methotrexate [7,15-17,19,21-24]. The most common regimen was a combination of carboplatin and paclitaxel. Treatment involved treating the underlying malignancy and then addressing PCD via immunomodulating therapy. Intravenous immunoglobulin (IVIg) (40%) [7,17,19,21,24,25] and corticosteroids (35%) [17,19,21,25] were the most common treatments used for managing PCD symptoms. Other immunosuppressants utilized for managing PCD symptoms were tacrolimus (15%) [16,21] and plasmapheresis (15%) [21]. Inhibition of extracellular signal-regulated kinases (ERK) was also utilized in the treatment of cancer at a rate of 5% [21], and 10% of studies reported no treatment beyond primary tumor resection [4,18]. While managing PCD and cancer symptoms, there was variability in the duration and dosing of treatments. A few studies noted the pairing of common treatment measures. For example, in the study by Renjen et al. (2018), carboplatin, paclitaxel, IVIg, and plasmapheresis were used as combination therapy [24]. IVIg was not used as a standalone treatment in any of the studies. Additionally, IVIg was not recommended in one of the studies due to a lack of adequate evidence [18]. Only 15% of studies used chemotherapy as a standalone treatment [15]. Treatment effects were highly variable and ranged from complete remission to no remission, but no definitive statements could be made because no studies compared therapeutic efficacy between agents, as they used either a single agent or a combination therapy.

Prognosis

Fourteen studies described the long-term effects among OC patients with PCD (n=20) [4,7,14-25]. Among these studies, there were four subgroups: patients who recovered from neuropathy (45%), patients with impairment halted at tumor resolution (15%), patients with progressive neurologic impairment (15%), and patients who succumbed to their condition (15%) [4,7,14-25]. Another key prognostic factor was the presence of anti-Yo, which was evaluated as an indicator of treatment efficacy [22]. However, positive anti-Yo in the serum or CSF was present in all four subgroups [16-23,25]. Anti-Yo antibodies were not detectable in both recovery instances and cases where PCD progression occurred at tumor resolution [4,7,15,25]. In the group of patients that did achieve symptomatic recovery, we found that dysphagia and simple speech progression were restored first, followed by lower limb motor strength, upper limb motor strength, and eventually gait restoration [7,17,18,20,22]. Neurological impairment can continue to worsen and eventually be the cause of death despite treating PCD [19].

Genetic Predisposition and Protein Expression

Pathophysiology for OC and Yo-PCD was discussed in four studies (n=81), exploring two different hypotheses: regulation of protein expression of onconeural antigens (66%) [26-28] and genetic predisposition through HLA alleles (33%) [29]. Raspotnig et al. (2017) examined sera samples from 16 patients with OC via western blot and immunohistochemistry [26]. CDR2 and CDR2L strongly stained the cytoplasm of cancer cells; however, only CDR2L strongly stained the cytoplasm of Purkinje cells [26]. Additionally, Vialatte de Pémille et al. (2018), in a study comparing the transcriptomic profile of 12 OC patients with PCD against 733 control patients with OC, noted that the gene CDR2 was downregulated and CDR2L was upregulated [28]. There was evidence to support that the differentially expressed genes were statistically significant in their correlation to cerebellar structures (family-wise error rate: <0.05) [28]. Small et al. (2018) collected data from 26 OC patients with anti-Yo antibody PCD and compared it against 116 control samples of OC that lacked anti-Yo antibodies [27]. It was found that chromosomal gain at 17q, the location that carries CDR2L, was significantly seen more in PCD patients (58.5%) than in control patients (30.1%) and had the highest concentration of differentially expressed genes [27,28]. Additionally, higher expression levels were seen for CDR2L; CDR2 did not show significant gains and had weak expression [27]. CDR2 and CDR2L genes in PCD patients frequently presented at least one somatic mutation (65%) [27]. Immune infiltration was higher in PCD tumors versus the control and more so in PCD preceding the tumor (p<0.05) [27]. A positive correlation was found between CDR2L density and immune infiltrate density [27]. Among the infiltrates observed were IgG-producing cells, plasma blasts, CD8+ T cells, regulatory T cells (Treg), monocytes, and naïve B cells [27,28]. Flow cytometry revealed a significantly higher infiltration of IgG-producing cells than in control tumors [27]. Additionally, CD8+ T cells were found in proximity to apoptotic tumor cells [27]. Upregulated autoimmune regulator (AIRE) genes (log fold change: 1.62, p<0.001) were found in OC samples with PCD, and 13% of other differentially expressed genes were supplemented with AIRE-related genes [28]. Hillary et al. (2018) performed high-resolution HLA typing and genome-wide association studies (GWAS) on 27 patients with OC, ataxia, and Yo-PCD and nine patients with breast cancer, ataxia, and Yo-PCD, and matched controls [29]. The HLA class II DRB1*13:01~DQA1*01:03~DQB1*06:03 haplotypes showed increased susceptibility to Yo-PCD in OC and breast cancer [29]. The DRB1*13:01~DQA1*01:03~DQB1*06:03 haplotypes' susceptibility to Yo-PCD was strongest within OC with a 33% incidence rate (9 of 27 patients) vs. only 9% in control patients (11 of 124 patients) [29].

Discussion

This systematic review sought to establish that the pathophysiological development of PCD could be multifactorial, with variations in individual HLA profiles leading to genetic susceptibility and immune tolerance breakdown tied to onconeural antigen differential expression. A combination of diagnostic tests should be considered to determine whether a patient exhibits symptoms related to PCD, including a clinical evaluation involving neurological assessment, appropriate imaging studies (to identify the primary tumor and stage), laboratory evaluation including serology, and genetic screenings. As indicated by the results, physicians may consider a multimodal treatment strategy that addresses the underlying malignancy and PCD symptoms. This review aims to highlight the latest options for identifying and treating PCD within the context of prevalence and pathology.

PCD is often a diagnosis of exclusion due to the lack of certainty regarding patient history, imaging results, and diagnostic evaluation [15]. A diagnosis of PCD may be suggested if the following criteria are met: a cancer diagnosis within five years of the onset of neuropathy, the presence of cerebellar symptoms characteristic of PCD, and the exclusion of other diagnoses causing the cerebellar symptoms [7], such as demyelinating diseases, atypical infections, systemic autoimmune disorders, medication toxicities, vitamin deficiencies, alcoholism, immune-mediated non-paraneoplastic causes, metastatic disease, and hereditary predisposition when presented alongside subacute ataxia [24]. The initial neurological assessment is not sufficient for a conclusive diagnosis of PCD, but additional imaging, serology, and a lumbar puncture can help narrow the differential diagnosis. Diagnostic imaging, including CT and MRI, can identify and stage the primary tumor and help rule out certain other etiologies for cerebellar degeneration [4,15].

Six antibodies hold significance in a PCD diagnosis from patients’ serum and CSF: anti-Yo, anti-Hu, anti-Ri, anti-amphiphysin, anti-CV2, and anti-Ma2/TA. Of these, anti-Yo antibodies have a higher propensity to reverse neurological symptoms, though this association is not necessarily correlated [18]. Cui et al. (2017) report that 40% of patients showed no observable antineuronal antibodies in serum or CSF paraneoplastic antibody panels [17]. These antibodies, notably anti-Yo, appeared in 14 patients (70%) of the subsample observed for long-term prognosis, of which eight displayed gradual recovery of neurological function or delay in the progression of neurologic impairment [18]. Despite this highly multivariate diagnosis, treatment of the underlying tumor holds precedence before the management of cerebellar symptoms [4]. Treatment of the tumor and timeliness of a diagnosis can be indicative of a stable neurologic outcome and less-severe cerebellar damage. This analysis of PCD studies provides evidence that early diagnosis in combination with therapies targeting both primary malignancy cancer and PCD led to better outcomes in reducing tumor progression as well as controlling neurological symptoms [19]. Lehner et al. (2021) conducted a study involving five patients with OC who received tumor-modulating therapy (n=5), steroids (n=5), IVIg (n=3), tacrolimus (n=3), and plasmapheresis (n=2) [21]. IVIg and corticosteroids were the two most common immunomodulating agents. In patients with PCD, timely diagnosis of the primary tumor and appropriate institution of antitumor therapy and specific PCD-directed therapy can improve neurological outcomes [4].

Although the complete pathophysiology of PCD development has not been established, there have been several hypotheses exploring potential mechanisms of breakdown at different levels. Many autoimmune diseases have been associated with certain HLA haplotypes that increase susceptibility to developing that disease. The findings from a recent study exploring HLA association with PCD suggest it follows a similar pattern. The increased prevalence of the HLA class II haplotypes DRB1*13:01~DQA1*01:03~DQB1*06:03 in OC patients with Yo-PCD could be a predisposing factor for PCD development [29]. HLA class II receptors from antigen-presenting cells (APCs) bind, process, and present extracellular antigens to CD4+ T cells to activate them toward those extracellular antigens [30]. In the case of Yo-PCD, certain HLA class II receptors created from the susceptible haplotypes perhaps have an augmented response to the onconeural antigens resulting in the activation of CD4+ T cells and B lymphocyte production of anti-Yo. Also, CD4+ T cells can prime CD8+ T cells to target specific antigens, such as onconeural antigens [31]. This process significantly amplifies the cytotoxic immune response as CD8+ T cells are believed to be the final effectors of Purkinje cell death [32]. However, HLA haplotype susceptibility alone does not account for all patients with OC and Yo-PCD since the haplotype prevalence within this subpopulation is lower than anticipated (33%). It is very likely that the HLA association only plays a small role in the larger immune tolerance breakdown associated with the pathophysiology behind PCD.

The HLA complex is one of many regulatory mechanisms involved in the maintenance of what is known as immune tolerance. This concept refers to the careful selection of immune cells that are sensitive enough to detect foreign antigens to mark for destruction while also recognizing self-antigens to protect against autoimmunity [33]. The AIRE gene encodes one of the most important proteins for the primary negative selection of T cells in the thymus for maintaining central immune tolerance [33]. Deficiency or mutation in the AIRE gene can lead to an improper presentation of self-antigens and can also lead to problems with autoimmunity [33]. A study exploring the transcriptomic profiles of several OC patients with PCD found that AIRE genes were upregulated along with CDR2L genes [28]. The latter finding is consistent with the other studies [26,27]. Remarkably, the AIRE gene is upregulated contrary to expectations of either neutral or downregulation in the case of PCD’s association with autoimmunity; upregulation could be potentially due to a variety of factors, including epigenetics and crosstalk among molecular pathways. In PCD patients, compared to controls, the CDR2L gene was more likely to be upregulated, mutated, and expressed at a protein level while CDR2 was differentially expressed and mutated while no significant changes were noted at a protein expression level [27,28]. Antigen presentation is a key part of positive and negative selection, increased prevalence of onconeural antigens may influence this process, while mutations common in CDR2 and CDR2L may provide an opportunity for genetic screenings. Screenings can be implemented via targeted next-generation sequencing of ovarian tumor samples [27].

Anti-Yo antibodies have previously been thought to contribute to the direct pathogenicity of Purkinje cells through some form of immune cell migration and infiltration [29]. Interferon-gamma (IFN-𝛾) is a cytokine that may stimulate a pathway that helps CD4+ and CD8+ T cells to migrate to the cerebellum. This cytokine is often present in high concentrations in the CSF of Yo-PCD patients [32]. Infiltration appears to begin at the tumor site with local differentiation of B cells as an indicator; the presence of naïve B cells, plasmablasts, plasma cells, and memory B cells is evidence of this differentiation [27]. The lymphocytes found in OC samples would support that of an acquired immune response. As stated, CD8+ T cells were in close proximity to apoptotic OC cells, which suggests an antitumor immune response [27]. Disrupting these key immune infiltrates could be helpful in the conversation about immune tolerance and the role of CD4+ cells in initiating this tolerance [26]. Treg cells are thought to also contribute to immune tolerance, as they act to suppress antitumor immune response [34]. When initiating immunotherapy, combination therapy may be more conducive to the permanent discontinuation of PCD symptoms, considering the redundancy in molecular pathways [35].

There has been a recent interest in understanding the mechanisms of PCD because novel cancer immunotherapies often target immune regulation checkpoints to enhance CD8+ T cell activity in killing tumor cells. The inactivation of CTLA4, an important immune checkpoint for the downregulation of CD8+ T cells, has been found to induce PCD and Purkinje cell death in mice [36]. Drugs like monoclonal antibodies that target the PD-1 pathway, another immune checkpoint, are already employed as treatments for certain types of cancer [37]. This therapy has often shown promising results in the regression of cancer, but it also carries an increased risk of autoimmunity if non-cancerous tissue expresses a tumoral antigen, such as in the case of OC with Yo-PCD. When screening for PCD, we can use genetic screens to confirm our clinical diagnosis with the support of patient history, physical exam, serology, and imaging. These genetic changes could be targeted with immunomodulating therapies.

Limitations

This systematic review was limited by the heterogeneity of the included studies, and hence a meta-analysis was not performed. The number of studies reviewed was small due to the rarity of adjunct PCD and OC. The limited sample size of this review may have created a risk of bias. Since the pathophysiology of PCD is not clearly known yet, there was some contradictory information in various reports, making it challenging to synthesize information. When reviewing studies for inclusion, studies at times did not distinguish PNS from PCD or present data exclusively on one type of cancer, which restricted the number of studies included in this review.

## Conclusions

Paraneoplastic cerebellar degeneration is a unique PNS that is thought to occur due to autoimmune destruction of the cerebellum and is most commonly seen in breast and pelvic malignancies; it tends to begin months or years preceding the cancer diagnosis. Timely recognition and removal of the primary malignancy are critical for overall clinical outcomes concerning patient health and cancer therapy effectiveness. We recommend that PCD diagnosis be considered if the patient has a history of malignancy within the past five years and has new cerebellar symptoms and no significant findings on imaging (ruling out other diagnoses). We can then confirm these suspicions with CDR2 and CDR2L antigen expression and genomic alterations and the presence of the anti-Yo antibody. Recognizing the symptoms of PCD and ordering the appropriate genomic and proteomic testing may facilitate early diagnosis and, consequently, an improvement in prognosis. Antitumor therapy and immunomodulating drugs are the most prominent therapeutic approaches supported by our findings; however, additional research is required before conclusive treatment recommendations can be made. By employing clinical judgment in conjunction with genetic and proteomic markers to diagnose PCD, clinicians may be able to detect malignancies sooner and prevent metastatic disease.
